# A new method for testing reproducibility in systematic reviews was developed, but needs more testing

**DOI:** 10.1186/s12874-021-01342-6

**Published:** 2021-07-29

**Authors:** Dawid Pieper, Simone Heß, Clovis Mariano Faggion

**Affiliations:** 1 Institute for Research in Operative Medicine, Faculty of Health, School of Medicine, Witten/Herdecke University, Ostmerheimer Str. 200, 51109 Cologne, Germany; 2 Department of Periodontology and Operative Dentistry, Faculty of Dentistry, University Hospital Münster, Münster, Germany

**Keywords:** Systematic reviews, Reproducibility of Results, Methodological quality, Data extraction, Risk of bias, Information storage and retrieval

## Abstract

**Background:**

To develop and test an approach to test reproducibility of SRs.

**Methods:**

Case study. We have developed an approach to test reproducibility retrospectively while focusing on the whole conduct of an SR instead of single steps of it. We replicated the literature searches and drew a 25% random sample followed by study selection, data extraction, and risk of bias (ROB) assessments performed by two reviewers independently. These results were compared narratively with the original review.

**Results:**

We were not able to fully reproduce the original search resulting in minor differences in the number of citations retrieved. The biggest disagreements were found in study selection. The most difficult section to be reproduced was the RoB assessment due to the lack of reporting clear criteria to support the judgement of RoB ratings, although agreement was still found to be satisfactory.

**Conclusion:**

Our approach as well as other approaches needs to undergo testing and comparison in the future as the area of testing for reproducibility of SRs is still in its infancy.

## Introduction

Systematic reviews (SRs) are essential to inform evidence-based decision making in health care across different groups such as clinicians, patients and policy makers. Despite this huge importance and potentially resulting implications for patients-related outcomes, it has been argued that currently there is a massive production of unnecessary, misleading, and conflicted systematic reviews and meta-analyses [1]. Among others, the Lancet series *reducing waste in research* recommended research studies to undergo rigorous independent replication and reproducibility checks [2]. In short, replication means that independent people will collect new data, while answering the same question. In contrast, reproducibility means that independent people will analyze the same data [3]. Given the definitions of replication and reproducibility from above, it becomes clear that replicability should be the ultimate goal and can be regarded to be placed over reproducibility. However, full and independent replication might not be feasible due to resource constraints. In this case, reproducibility can be seen as a way to serve as a minimum standard for judging scientific claims [4].

It was found that reproducible research practices are uncommon in SRs, and thus limiting the possibility of testing for reproducibility [5]. Others dealt with single steps of conducting SRs. For example, studies found the reproducibility of search strategies to be poor [6, 7]. Others indicated that study selection, data extraction, risk of bias assessments and meta-analyses might also lead to different results depending on the author group involved [8–11]. This implicates that these steps might also be difficult to be fully reproducible. Gaps in reproducibility in several steps of a SR potentially results in a lack of replicability.

Some first ideas have been presented how testing for reproducibility in SRs could work [12]. However, to the best of our knowledge no testing of the whole SR instead of single steps has been conducted. Therefore, we set out to develop and execute a strategy to test for reproducibility in a SR. Our strategy comprised the reproducibility of the following steps of a SR: search, selection, data extraction and risk of bias (RoB) assessment.

## Methods

The methods section is divided into two parts. The first part (2.1) describes our developed idea for proportional testing for reproducibility in systematic reviews (PTRSR). This approach is tested on a single SR. This is described in the second part (2.2).

### Proportional testing for reproducibility in systematic reviews (PTRSR)

One of the main ideas of the PTRSR is that it can be conducted at any time after a SR has been published (retrospective). This will allow for testing older SRs for reproducibility as well. At the same time, more than one reproduction of a SR can be conducted (e.g. by several author groups), and thus giving more power to the reproducibility test, when assuming that they come to the same result. Other approaches to test reproducibility could also include prospective elements (e.g. two independent pairs of researchers working in parallel).

The general idea of the PTRSR is that the formerly published SR is not reproduced in full, but only for a given proportion of it. This might increase feasibility given that obtaining funding and being rewarded in any way might be difficult to achieve. According to Page et al. 2016 a therapeutic non-Cochrane SR includes a median of 14 included studies [13]. Thus, we suggest starting with a 25% proportion test, i.e. only 25% of the SR will undergo the reproducibility test. This would result in approximately 3.5 studies per SR what we have considered to be the minimal value allowing for a meaningful test. However, this is an arbitrary choice. This number needs to be adjusted when the SR does only include few studies. It should be noted that the 25% do refer to the number of hits obtained from the literature search, but not to the finally included number of studies.

In a first step, the reproducibility team (RT) will conduct all searches in bibliographic databases. After having merged all search results into one database the RT will compare their number of hits with the number of hits in the original review. If no major disagreements will be identified at this stage, the RT will draw a 25% random sample of all identified hits. After that, all forthcoming steps will be performed in the same way as reported in the original SR (see Table 1).

**Table 1:** Stepwise approach for applying the reproducibility concept to systematic reviews

Step 1 (obligatory): replicate all searches in bibliographic databases and combine them in one database
Step 2 (obligatory; percentage can be increased, e.g. in case of review including a small number of studies): draw a 25% random sample
Step 3 (obligatory): perform study selection (title&abstract and full-text) in the same way as reported, and applying the same criteria as reported in the original SR
Step 4 (obligatory): extract data for the main outcomes (e.g. primary outcomes, outcomes shown in the main text only (i. e. excluding supplementary materials))
Step 5 (obligatory): assess risk of bias/methodological quality as in the original SR
Step 6 (optional^a^): perform evidence synthesis as in the original SR. This might include meta-analyses (also including studies found to meet eligibility criteria not included in the original review) and applying systems for assessing the quality of evidence such as GRADE, for example

^a^ step 6 is optional as this is likely to need another approach than using the 25% sample

Results (i.e. the comparison of the original SR with the reproduced SR) can be categorized for different steps depending on their importance for reproducibility. In other words, different results can occur for some steps of the SR process without decreasing the overall confidence in the findings of the SR. For example, the number of full-texts to screen (i.e. abstracts that met eligibility criteria at title & abstract screening) is likely to vary between different research groups as some research groups might be more inclusive than other. However, no differences should be expected for the included studies, when clear eligibility criteria are reported. All relevant studies need to be included in both SRs. All studies from the original SR should be included by the RT, while at the same time the RT should not identify more eligible studies than in the original SR.

### Case Study

We wanted to test our developed approach (2.1) on one SR (case study). Given that we have developed the approach, we might have been biased in choosing a SR. Therefore, we reached out to an information specialist and asked her to provide us a SR eligible for this case study. The information specialist was blinded against the aim of our study. The eligibility criteria were developed against the background that only well-reported SRs will qualify for undergoing testing reproducibility as outlined in 2.1 (Table 2).

**Table 2:** Eligibility criteria for choosing a SR for our case study

• SR on a healthcare intervention including only randomized controlled trials (RCTs)
• Number of included studies ≥ 50 (to make sure that enough studies will be included and the reproducibility test will be completed successfully)
• Search strategies reported for all bibliographic databases
• Providing a full list of references of all included studies
• Reported risk of bias assessment
• Meta-analysis for at least one outcome

With these criteria, the information specialist identified a SR entitled “Effects of omega-3 polyunsaturated fatty acid intake in patients with chronic kidney disease: Systematic review and meta-analysis of randomized controlled trials” (thenceforth labelled “original SR”) published online in 2019 that was chosen for our case study [14]. This review included sixty trials with 4,129 participants, searched several databases, performed risk of bias assessment applying the Cochrane risk of bias tool, and performed several meta-analyses including subgroup analyses.

We followed our stepwise approach (Table 1) from step 1 to step 5. Results for each step contain the comparison between the original SR and the RT. Data are presented quantitatively and discussed for each step.

## Results

### Searches in bibliographic databases

The original review searched MEDLINE, EMBASE and CENTRAL. The full search strategies were provided in the supplementary material. Time restrictions were also reported together with the number of hits obtained in each bibliographic database. All searches were restricted to the same end data in order to correspond to the original review. For all three databases, we were not able to get exactly the same results. For MEDLINE, we copied the complete search strategy. We used Ovid as an interface, while the interface was not explicitly reported in the original review. However, after having investigated the search string we came to the conclusion that it was very likely that Ovid was also used in the original review. While the original review retrieved 518 hits, we were only able to retrieve 462 or 499 (searching without any time restriction) hits. Also for CENTRAL, the complete search strategy was copied and re-run. Again, the result was not fully reproducible. We retrieved fewer hits than the original review did (692 vs. 721). We have no explanation for this difference. The biggest difference between the original review and the RT was found for EMBASE (142 vs. 86). This difference might be explainable by many factors. First, we had to adapt the literature search from the Ovid to the Elsevier interface. Two text descriptors (*tw* and *sh*) used in the original research were not available when searching via the Elsevier interface. Both were replaced by the descriptors *ti, ab, kw*. It was also not clear whether the authors of the original review applied any restrictions to the publication type.

**Fig. 1:**
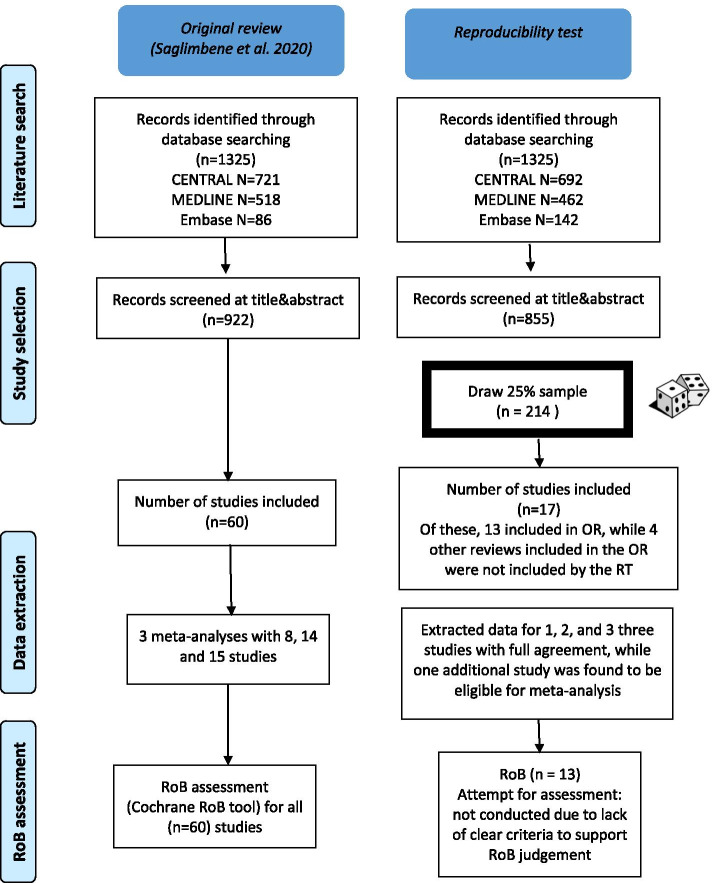
Comparison of the original review with the results obtained by the reproducibility team. RoB risk of bias, OR Original review, RT reproducibility team

In total, after removing duplicates 922 hits were screened at title&abstract level in the original review. All but one hit were identified through the searches in bibliographic databases. One hit was identified through other sources. In contrast, our combined searches retrieved 855 hits after removing duplicates (Fig. 1).

### Study selection

After drawing a random 25% sample, 214 citations were screened. Of those, 42 were found to be potentially relevant, and the full-text was retrieved. The RT included 17 articles meeting the eligibility criteria. Only 77% (13/17) of the full-texts included by the RT were also found to be included in the original review. No obvious reasons were found to explain the disagreement. There were also four other articles that were included in the original review, while they were found not to meet the eligibility criteria by the RT. All four articles were excluded at full-text level. Again, these disagreements could not be explained. No articles were excluded by the RT at title&abstract level that were included in the original review.

### Data extraction

To check the reproducibility of data extraction, the RT checked whether the 13 studies they have found to be eligible and that were also included in the original review contained data relevant for meta-analysis. As several meta-analyses were reported in the original review, we decided to focus only on three meta-analyses. These meta-analyses focused on primary outcomes and were all reported and shown with a forest plot in the main article. In the case that a study was found to be eligible for meta-analysis we extracted all relevant data for the meta-analysis, i.e. number of patients and number of cases in each arm for dichotomous outcomes, and mean, standard deviation and number of patients in each arm for continuous outcomes. The meta-analyses included eight, fifteen and fourteen studies in the original review, respectively. From the 25% sample one (13%), two (13%) and three (21%) studies were included in the original review, respectively. All of these studies were identified by the RT. Furthermore, all extracted data fully matched the data in the meta-analysis in the original review. However, we found one additional study that should have been included in the second meta-analysis (without having affected the result). This was the only disagreement in this context.

### Risk of bias assessment

The RT performed RoB assessment of the 13 included RCTs. To reproduce the rating we first checked whether the authors reported the criteria used in the original SR to support the judgements of ROB. Because the SR authors did not clearly reported these criteria, we were only able to compare the ratings without taking any rationale into consideration. Two reviewers (DP, CF) of the RT independently performed RoB assessment following the guidance in the Cochrane handbook. In a first step, we calculated raw agreement within the RT. In a second step, we compared the results of the RT against the ratings in the original SR. Four outcomes were possible at this stage: full agreement between RT and original SR (1 point), partial agreement between RT and original review (i. e. only one rater agreed with the original SR; 0.5 points), no agreement between RT and original SR (0 points). In the last case, we also aimed to differentiate whether there was agreement within the RT or not). However, there was no case of disagreement between the RT and the original SR where the RT would have also disagreed. The raw agreement from 0.65 to 0.92 and 0.38 to 0.92 depending on the outcome domain (Table 3) within the RT and between the RT and the original SR, respectively. Among the 26 disagreements among the RT only 6 disagreements were opposite (i.e. high vs. low RoB).

**Table 3:** Agreement in risk of bias assessment

	Random sequence generation	Allocation concealment	Blinding participants or personnel	Blinding outcome assessors	Incomplete outcome data	Selective reporting	Other Bias
Reproducibility team (within)	0.85 (11/13)	0.92 (12/13)	0.75 (15/20)	0.65 (13/20)	0.75 (15/20)	0.69 (9/13)	0.85 (11/13)
Reproduction team vs. original SR	0.92 (12/13)	0.88 (11.5/13)	0.48 (9.5/20)	0.78 (15.5/20)	0.38 (7.5/20)	0.77 (10/13)	0.62 (8/13)

## Discussion

To the best of our knowledge, this study is the first one to investigate the reproducibility of a SR that does not focus only on single steps, but on the whole SR. Our developed strategy to test reproducibility was based on a 25% proportion of the original review. In all investigated steps of literature search, study selection, data extraction and ROB assessment we found reproducibility to be satisfactory although some issues remained unexplained.

We were not able to fully reproduce the number of hits obtained from searching bibliographic databases. This is in line with former studies on this topic [6, 15]. To a larger extent, we found differences in study selection, including studies that should have been included according to the eligibility criteria, while other should not be included. We found only one disagreement in the extracted data that was unlikely to have an impact on the review´s conclusion.

Our randomly chosen SR will probably not reflect the average reporting and methodological quality of SRs in biomedical literature. We applied rather strict to eligibility criteria. This was necessary to secure the feasibility of our approach. The feasibility already starts with an adequate reporting of search strategies. In a sample of SRs in the field of anesthesiology only 10.5% of all SRs presented a full search strategy. Of those, only 57.4% reported the full search strategies for all sources [15]. An investigation in high impact journals found that 22% of articles provided at least one reproducible search strategy and 13% allowed reproducibility for all databases searched [6]. Older studies showed even less satisfying results [16, 17]. This might indicate that earlier calls for better reporting of search strategies have been heard [18], and reporting is improving, and thus facilitating more reproducibility checks in future. It is also important to note that we focused only on reproducing electronic searches for several reasons. Conference abstracts might be difficult and expensive to obtain if not available in an electronic format. Google scholar cannot reproduce results by definition [19]. Searching for grey literature might also be hardly reproducible. However, our choice was not only informed by pragmatism but also by evidence. Prior studies even found that searching electronic databases beyond PubMed does not lead to substantial changes in the results of SRs [20, 21]. The impact of searching grey literature may also be considered to be low, although this might be dependent on some factors such as the topic under study or the few number of studies included [21–23].

There is much less literature on study selection errors in systematic reviews. One small study estimated that pair of reviewers missed 4% of potentially relevant records, i.e. comparing pair of reviewers to the original review at title&abstract level [24]. Ford et al. replicated eight SRs of pharmacological interventions for irritable bowel syndrome and found that six meta-analyses missed 17 separate RCTs, constituting 3–11% of eligible patients not been included [25]. However, the authors did not investigate whether the reason for missing these RCTs could be attributed either to study selection or literature search.

Our result for data extraction was very satisfactory. Other studies also indicated that data extraction errors occur regularly leading to biased estimates [26–28], while this will usually not impact the conclusions of the SR [29, 30]. One exception was the extraction on RoB ratings from the original publication. There was limited and unclear information on the criteria used by authors to rate RoB. The full report of the rationale used to judge ROB should be a standard in any SR to allow any kind of auditing purposes. According to MECIR 2021, the rationale for RoB ratings should be reported in Cochrane intervention reviews [31]. However, this has already been discussed earlier, and should become standard in all SRs [32]. Given that usually supplementary materials can be made available online this should not be a challenge. Nevertheless, although there were disagreements between the RT and the original SR, the agreement can still be regarded to be quite satisfactory when compared to studies investigating RoB agreements between SRs [8, 9].

Overall, the first three steps (Table 1) of our approach can be well anticipated in advance. In particular step 4 (data extraction), but also steps 5 and 6, cannot be anticipated in the same way as they heavily depend on the number of included studies, outcomes and performed analyses. Thus, it is much more difficult to provide clear guidance for these steps. For example, if only one meta-analysis was performed it is a clear-cut choice that data extraction should focus on it, while the choice might be less obvious in case of multiple meta-analyses. Authors should clearly report and explain their choice for these steps.

The completeness of reporting the SR steps seems pivotal to achieve full reproducibility. For example, this is the case in the step of study selection results from the literature search. Replicating this step will hardly be ever possible. The authors would need not only to report the list of excluded studies at full-text level what is in accordance with the *Preferred reporting items for systematic reviews and meta-analyses* (PRISMA) guideline [33], but start to present a full list of all citations retrieved at title&abstract level. The steps of data extraction and RoB assessment could have been conducted independently of the others as all included studies were reported. The outcome data to retrieve were quite straightforward in our case study. We acknowledge that this is dependent on the type of data (e.g. binary vs. continuous, number of measurements (different time points)), and larger discrepancies can also be expected [34–36].

We understand that the report of every step in a SR should be complete enough to avoid any further contact with SR authors to clarify potential issues. Further contacts would imply in lack of efficiency in the use of resources, and we feel it is not the responsibility of assessors to check data beyond of what is reported in the scientific article. In fact, we understand that it is the obligation of SR authors to provide full report to allow the reproducibility of the steps. One important question here is: how to be certain that answers provided by authors are in fact accurate? Some evidence suggests that contacting authors can modify important outcomes of SRs [37]. Thus, should we trust in the results of SRs where authors were not contacted to provide extra information?

In psychology, a new article type called Registered Replication Reports has been introduced by the journal *Advances in Methods and Practices in Psychological Science*. The RTs can submit a detailed protocol for an ongoing study. This protocol is then forwarded to the authors of the original study for feedback. In contrast to our approach, RTs already submit their plan prior to data collection of the original study. This comes along with two potential advantages. First, the result of the RT will be published irrespective of the result, and thus giving credit for their effort. Second, the described process aims more for constructive feedback than for identifying errors in others work [38]. Such an approach could potentially also be feasible for SRs in biomedicine. However, the relatively short time period between registering a review or publishing its protocol and start of data collection would give RTs only a very limited time window to submit their reproducibility proposal.

Overall, it becomes obvious that both, reporting quality and methodological quality of a SR play a crucial role, and this needs also to be kept in mind when choosing SRs for undergoing a reproducibility test. We argue that applying our approach to low quality SRs might be useless Furthermore, critical flaws in the methods (e.g. wrong statistical methods) can be a reason not to test for reproducibility at all. Tools for assessing the methodological quality of SRs such as AMSTAR 2 [39], for example, can be applied to identify critical flaws. In some cases, however, a meta-analysis should be reproduced or conducted again in an adequate way to obtain more accurate results or clarify conflicting meta-analytic results from different meta-analyses on the same research question.

## Limitations

Our randomly chosen SR focused on a health care intervention and included only RCTs. Although this is the most frequent type of a SR [13], we admit that our approach would possibly need modifications for other types of reviews (e.g. diagnostic test accuracy reviews). In general, more methods studies focusing on the single steps of conducting SRs are highly appreciated to substantiate evidence for and reproducibility testing. A recent review revealed a lack of such studies [40]. We felt that the steps of literature search, study selection and RoB assessment were sufficient to gain knowledge of the underlying steps. When searching literature, we were not always able to use the same interfaces either due to non-reporting in the original SR or because of having no access to the interfaces used. However, we only extracted data for meta-analyses. Given the aforementioned evidence on errors in meta-analyses it might also have been interesting to reproduce one meta-analysis in full. Our approach lacks of formalized overall assessment of the PTRSR. We believe that more practical tests are needed to test the feasibility and applicability of our approach first to confirm that the domains are chosen and operationalized correctly. Last, we did not get in contact with the authors of the original review. We also have to acknowledge that the RT had no content expertise in the review under study. However, as we did not interpret the findings to come up with clinical recommendations, we think that this might be a negligible factor. Furthermore, it has been formerly argued that content expertise might not be that important for authors of SRs [41].

## Conclusion

Our approach resembles a post-publication review that is performed in a structured way. Thus, reproducibility tests can become a part of such post-publication reviews and allow the original review authors to improve on their review in terms of reporting and methodological quality [42].

An essential step in reproducing SRs is that SR authors make all of their data accessible. This will allow reproducibility and increase the credibility of SRs [43]. Our approach as well as other approaches needs to undergo testing and comparison in the future as the area of testing for reproducibility of SRs is still in its infancy.

## Data Availability

All research data are available upon request from the corresponding author.

## Abbreviations

RCT: randomized controlled trial
SR: systematic review
PTRSR: proportional testing for reproducibility in systematic reviews
RT: reproducibility team
RoB: risk of bias
OR: original review
PRISMA: Preferred reporting items for systematic reviews and meta-anaylses
